# The Easily Overlooked Effect of Global Warming: Diffusion of Heavy Metals

**DOI:** 10.3390/toxics12060400

**Published:** 2024-05-30

**Authors:** Wenqi Xiao, Yunfeng Zhang, Xiaodie Chen, Ajia Sha, Zhuang Xiong, Yingyong Luo, Lianxin Peng, Liang Zou, Changsong Zhao, Qiang Li

**Affiliations:** 1 Key Laboratory of Coarse Cereal Processing, Ministry of Agriculture and Rural Affairs, Sichuan Engineering & Technology Research Center of Coarse Cereal Industrialization, School of Food and Biological Engineering, Chengdu University, Chengdu 610106, China; xwq990713@126.com (W.X.); zhangyunfeng@cdu.edu.cn (Y.Z.); cxd0512@126.com (X.C.); shaajia19980108@126.com (A.S.); xiongzhuang2000@126.com (Z.X.); lyy1478963@126.com (Y.L.); penglianxin@cdu.edu.cn (L.P.); zouliang@cdu.edu.cn (L.Z.); 2 School of Public Health, Chengdu Medical College, Chengdu 610500, China

**Keywords:** global warming, heavy metals, soil pollution, marine pollution, ecological effect

## Abstract

Since industrialization, global temperatures have continued to rise. Human activities have resulted in heavy metals being freed from their original, fixed locations. Because of global warming, glaciers are melting, carbon dioxide concentrations are increasing, weather patterns are shifting, and various environmental forces are at play, resulting in the movement of heavy metals and alteration of their forms. In this general context, the impact of heavy metals on ecosystems and organisms has changed accordingly. For most ecosystems, the levels of heavy metals are on the rise, and this rise can have a negative impact on the ecosystem as a whole. Numerous studies have been conducted to analyze the combined impacts of climate change and heavy metals. However, the summary of the current studies is not perfect. Therefore, this review discusses how heavy metals affect ecosystems during the process of climate change from multiple perspectives, providing some references for addressing the impact of climate warming on environmental heavy metals.

## 1. Introduction

The climate is being affected by human activities; a phenomenon referred to as global warming. The burning of fossil fuels and large-scale deforestation have resulted in the emission of large amounts of “greenhouse gases” into the atmosphere, the most important of which being carbon dioxide [1]. These gases trap the infrared radiation from the Earth’s surface, creating a thermal barrier that furthers the greenhouse effect. During the 20th century, the global surface temperature increased by 0.8 °C, and the current global climate temperature is 1.1 °C higher than the pre-industrial global climate temperature [2]. It is estimated that the global climate temperature will rise by 1.4–5.8 °C in the 21st century [3].

The environment is the aggregate of external conditions that encompass a single organism or a collective of organisms and are essential for their growth, development and continued existence [4]. The impacts of global warming on the environment are multilevel and multifaceted. First, global warming itself is a change in climatic resource conditions; global warming will lead to increases in climate temperature and precipitation [5], changing the spatial distribution pattern of water resources, with some areas tending to be drier, while some areas will tend to be wetter. Second, global warming, on the one hand, melts continental glaciers in bipolar regions, and a large amount of glacial meltwater carries elements of each glacier into the ocean [6]; on the other hand, global warming is heating and expanding seawater, and the two together are promoting sea level rise, which is submerging coastal lowlands and some islands [7]. Third, global warming will cause changes to the Earth’s original ecosystems [8]. The physical geographic environment has a holistic character and physical geographic elements, such as topography, climate, biology, hydrology, and soil, affect each other. Therefore, changes in climatic conditions will cause changes in the entire ecosystem. In general, the effects of global warming are multifaceted, with impacts on the distribution of natural elements, productivity, natural ecosystems, agriculture, rangelands, forestry, and society [1,9,10,11,12].

“Heavy metal” refers to any heavy metal element with a relatively high density [13] and is a suitable term for heavy metals and heavy metalloid groups with an atomic density greater than 4 g/cm^3^. Heavy metals pose a major threat to the environment, and their toxicity is becoming an increasingly urgent concern due to its implications for the ecosystem, evolution, nutrition, and the environment. Even at low concentrations, they are toxic or harmful [14,15], and the accumulation of heavy metals in soil is a concern in agricultural production [16], which has an adverse effect on the environment of plants and their metabolic activities, as well as on soil organisms [17]. Heavy metals can enter the human body by way of the food chain. Within the body, these metals can interact with proteins and enzymes, leading to a decrease in their performance or causing them to become inactive [18]. Accumulation in certain organs of the human body can cause long-term poisoning. Heavy metals are distributed in the air, water, and soil, and therefore their impacts on the ecosystem and humans are multifaceted and multilayered [14,19,20,21,22].

The effects of global warming-induced heavy metals on ecosystems are multifaceted. Currently, a large number of studies both at home and abroad have reported these connections. This can help us address the adverse effects of heavy metals. Some articles have summarized climate-driven interactions between heavy metals and organisms [23]. Reports indicate that climate change will significantly influence the movement and activity of pollutants by changing the physical, chemical, and biological components that are distributed among the air, water, soil, sediment, and living organisms [24]. An exhaustive examination of research into heavy metal contamination in coastal wetlands worldwide has been made available [25]. However, articles summarizing the impacts of global warming-altered heavy metals on ecosystems are lacking or inadequately summarized. For the past ten years, investigations into the consequences of heavy metals on global warming have been on the rise. Therefore, this paper provides a more complete overview of the interactions between the migration and morphological changes of heavy metals on organisms, the environment, and humans under global warming conditions, and provides some references for subsequent research on heavy metals under climate change.

## 2. Transport of Heavy Metals in the Environment under Global Warming

**Figure 1:**
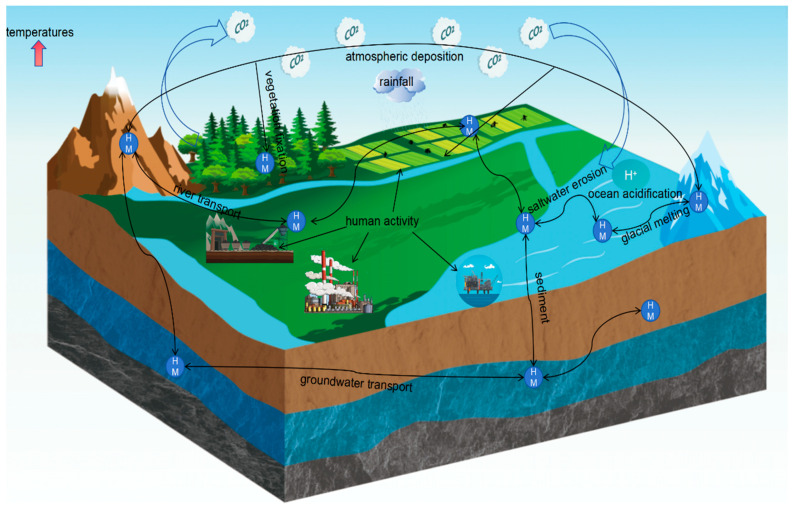
Migration of heavy metals in the environment.

Human activities such as mining and metal smelting, which release metals into the environment, also contribute to global climate change, which may affect the geochemical and biogeochemical cycling of heavy metals. As the climate warms, the temperature and humidity in the soil may change, which may affect the transport and transformation processes of heavy metals in the soil. For example, higher temperatures may accelerate the decomposition of soil organic matter, which may affect heavy metal adsorption and desorption processes. Climate change may also lead to changes in precipitation patterns, which may affect the leaching and transport of heavy metals, which may increase heavy metal concentrations in soils and water bodies. Warmer temperatures will also melt glaciers, and metal elements in glaciers will flow into the oceans. Metals can also be transported further into the environment through the atmosphere, rivers, and other pathways. These environments take up metals from these pathways through atmospheric deposition and plant fixation, causing an increase in metal levels in the environment. Overall, climate change enhances the mobility of metals in various ways. Figure 1 is a schematic representation of some of the climate-driven transport pathways for heavy metals in the environment.

### 2.1. Air

Human activities such as industrial emissions, transportation, and fossil fuel combustion are the main causes of global warming. Human activities such as industrial emissions, transportation, and fossil fuel combustion release large amounts of pollutants into the air, such as particulate matter (PM), carbon monoxide, nitrogen dioxide, and heavy metals [26,27]. Heavy metal elements are released into the atmosphere through exhaust emissions and incomplete combustion, posing a potential threat to human health and the environment. Heavy metals can diffuse to other areas through particle transport and gas flow [28]. Heavy metals in the atmosphere generally enter the ecological environment through sedimentation. Atmospheric deposition is divided into dry deposition and wet deposition [29]. Dry deposition refers to the relative movement of free-falling atmospheric particulate matter (PM) towards the Earth’s surface caused by direct collision or gravity deposition. Wet deposition refers to the process in which particles and gases dissolve or suspend in water droplets and then deposit to the ground in precipitation (such as rain, snow, etc.) [30,31]. Heavy metals can be absorbed and fixed by plants or combined with sulfides in the environment [32]. The bioavailability of heavy metals that settle from the atmosphere to the surface soil will be higher [33]. In addition, heavy metals in the air may also come into direct contact with organisms.

### 2.2. Aquatic Environment

Aquatic ecosystems will be affected by global warming in a variety of ways, such as melting glaciers, increased sea levels, more frequent storm events, ocean acidification, salinity changes, ocean temperature rises, and marine heat waves [34]. Global warming and the synchronous increase in glacial meltwater are the two main factors driving the input of natural elements from land into lakes and oceans, and certain human factors have also increased the input of heavy metal elements. Table 1 shows the changes in the heavy metal contents in some lakes and oceans around the world. The heavy metal contents in these lakes and oceans are constantly increasing.

With the world’s climate warming, glaciers are melting and thus releasing a substantial amount of metals previously trapped inside them into the oceans and other downstream ecosystems. This includes mercury from human-induced and natural sources, which are becoming mobilized from permafrost and other Arctic soils as a result of climate change [42]. It has been recorded that glaciers of 400 to 600 mg of Hg have accumulated in the setback area [43]. In the future, another 20% of Alpine glaciers or polar glaciers may melt due to rising temperatures [44]. There are many migration pathways of heavy metals, most of which flow into runoff and flow to downstream ecosystems [45]. One study used stable isotope techniques to show that vegetation is the main driver in immobilizing heavy metals released from melting glaciers. The heavy metals released into the atmosphere may be deposited in the surface soil through precipitation and plant uptake, and the heavy metals that are not adsorbed and fixed will eventually flow into the ocean.

The ocean’s seawater characteristics, including pH, salinity, and temperature, are altered by global warming, which in turn influences the leaching and transport of heavy metals [46,47]. Oil spills from ships and drilling platforms [48] and the leaching of tailings in the ocean increase the content of heavy metals in the ocean [49]. The heavy metal content of coastal wetlands is increased by human activities such as agriculture, aquaculture, and wastewater discharge [50], and it has been found that changes in precipitation will affect the mobility of Cd, Pb, and Zn in wetland soils. Therefore, changes in precipitation caused by global warming will increase this mobility, which may allow the heavy metals in the wetlands to flow to the ocean [51]. Marine organisms can also act as vehicles for the transfer of heavy metals, and marine plankton play a key role in the recovery and transportation of heavy metals such as mercury, cadmium, and lead in the food web [52]. Ocean current fluctuations are caused by global warming. To a certain extent, this change makes the contact area between plankton and the ocean larger and more extensive, and then heavy metals are released into more waters through food chain relationships or biological behavior [53].

Around 13–30% of the total freshwater is groundwater, supplying drinking water to more than half of the world’s people [54]. An increase in the level of heavy metals found in groundwater could lead to serious risks to human health and survival. The heavy metal content in groundwater is also affected by climate change. Research has employed a mixed effects model to demonstrate that human behavior and climate have a considerable effect on the diffusion of heavy metals in groundwater [55]. Bacteria and organic matter present in sediments have the capability of adsorbing heavy metals. When the temperature of the water rises, bacteria and enzymes in the sediment become more active, and under certain circumstances, heavy metals can be transformed into forms that are soluble in water and can be exchanged, thus being released into the water [56]. Elevated temperature can also regulate the kinetics of heavy metal precipitation or solubilization by affecting pH and redox potential [57]. The changes in climatic conditions caused by global warming change the hydrodynamic conditions of aquatic ecosystems; for instance, when precipitation increases, the flow rate of rivers increases, affecting the redistribution of heavy metals [58]. It may also cause the heavy metals in the sediment to migrate to the upper water layer and diffuse with the water flow.

### 2.3. Soil

Due to rapid industrialization and the intensification of human activities, soil is facing enormous pressure from many pollution sources. Heavy metals are of particular concern due to their capability to spread, their lack of degradation, and their potentially harmful impact [41]. Global warming causes an increase in temperature, which affects soil biological activity and physicochemical interactions. Heavy metals can migrate horizontally as well as vertically, and under physical, chemical, and biological effects, they can produce morphological changes and migrate to other media, and a variety of factors jointly affect the migration and transformation of heavy metals in the soil, thus ultimately changing the mobilization of soil trace elements [59].

Mineral resources are a key material basis for global socio-economic development [60], and although mineral resource mining is very important, it is also the main man-made cause of global warming. At the same time, it has caused a major issue of heavy metal contamination in soils all over the world, especially in the vicinity of heavy metal mining and processing areas. Among them, heavy metals can migrate from mines to farmland through various channels [61]. In particular, soil leaching or transport via surface runoff to rivers and then to downstream areas [62] leads to the accumulation of pollutants in the soil [63].

Global warming increases temperatures and precipitation, making it easier for heavy metals to migrate. Changes in temperature and changes in precipitation patterns alter soil water dynamics, and temperature rises and precipitation changes caused by global warming will affect the distribution of water and elements in the river basin and surrounding soils [64]. One study analyzed sediments from two watersheds in two different climatic zones and found that the proportions of acid-soluble components of Cu and Pb were higher in the subtropical watersheds (23.55–33.60%), suggesting that increased temperature accelerates the transformation of heavy metal components and promotes the transformation of heavy metals from a relatively stable fraction to a more unstable fraction [65,66], thus facilitating the transport of heavy metals [67]. Examining the black soil and seasonal snow cover of a typical farmland in Northeast China, a study was conducted to evaluate the effects of global warming. The results showed that climate warming had reduced the duration of snow melting, resulting in a decrease of heavy metal content in the snow; yet, the metals were entering the soil earlier. Heavy metal contents in the tillage layer of farmland soil showed an increasing trend, and the risk of heavy metal pollution increased [68], while crop quality and productivity decreased [69,70].

Groundwater and the soil system also influence one another. As sea levels rise due to climate change, coastal areas experience seawater erosion. Seawater intrusion may alter the characteristics of soil water systems [71]. The leaching of Fe and Mn from minerals is accelerated by brine intrusion, which increases the ionic strength and reduces the ionic activity coefficients, thus promoting the dissolution of Fe and Mn. Seawater erosion provides a stable channel for the natural elements in seawater to enter the terrestrial soil system [72].

### 2.4. Environmental Metal Risks

At present, the monitoring methods for heavy metal pollution in the environment are mostly biological monitoring, such as tree rings and moss [73,74]. Changes in heavy metal content in the ecological environment where trees are located can be inferred by detecting changes in the metal content in their annual rings [75]. The above studies have all reported that the metal content is increasing year by year. When calculating the critical load of heavy metals in a specific environment, existing research considers four aspects: metal toxicity, human exposure risk, environmental load, and environmental risk assessment [76,77].

Table 2 shows the levels of heavy metals in the environment in different regions, and in some areas the levels of these metals have exceeded the permissible limits set by some standards.

## 3. Heavy Metal Bioavailability under Global Warming

The bioavailability of heavy metals is closely related to the existing forms of heavy metals in the environment. When heavy metals are in a free state, it is easier for organisms in the environment to absorb and utilize them. Global warming can lead to changes in various environmental factors that can mediate changes in the morphology of heavy metals or enhance the binding of heavy metals between organisms and heavy metals, thereby altering the bioavailability of these metals.

### 3.1. Temperature

Heavy metals can move into the soil ecosystem and then be taken in by living things. The high availability of heavy metals and heavy metal-like substances threatens all biota more directly than their total content [101], and many factors affect their availability, such as temperature and organic matter content [102]. Some studies have reported that by the end of the 21st century, the global surface temperature will rise by 1 to 3.7 °C [103]. Climate change has been shown to alter soil properties, with warming increasing soil enzyme activity rates and soil respiration, accelerating the decomposition of organic matter and nutrient turnover and increasing plant growth and feedback of apoplastic matter to the soil [104]. Soil physicochemical properties, such as SOC, cations, and pH value, also change significantly as the temperature increases [105], which may further affect the availability of heavy metals. An increase in temperature may also affect the speciation of heavy metals. A study of groundwater samples collected from Alpine lakes for incubation experiments suggests that environmental changes caused by global warming provide conditions that promote mercury methylation and photodemethylation of MMHg [106]. Methylmercury is a strong neurotoxin. Warming gradually decreases the pH of soil pore water, increases the concentration of water-soluble Cd/Cu, and reduces the formation of iron spots on the surface of the plant root system, thus significantly increasing the total uptake of Cd/Cu by the plant [107]. Meanwhile, warming promotes the translocation of Cd from roots to aboveground and increases the percentage of aboveground Cd distribution, which adversely affects the safety of food [108]. Soil water content also affects the uptake of heavy metals by animals and plants. Temperature and precipitation affect the soil water content and temperature, which in turn affects the structure of soil aggregates and the growth of plants, thus influencing the activity of microorganisms in the soil [109]. Several studies have demonstrated that when air temperature and soil water content are combined, the bioaccumulation of heavy metals in animals and plants is higher in warm and dry environments [110,111]. Plant experiments showed that wheat growth and root uptake of copper are highly dependent on soil type but less affected by temperature. Therefore, the effects of global warming on different types of soil and the plants grown in them vary [105]. Temperature can influence the uptake and toxicity of heavy metals by changing the stability of adsorbed ions, increasing metabolic rate, disrupting mitochondrial function, causing oxidative stress, leading to the accumulation of lipid peroxidation products, damaging the lysosomal system, and damaging DNA [112,113,114].

### 3.2. CO_2_

The environmental changes caused by global climate change are mainly manifested as temperature increases and carbon dioxide concentration increases. The global atmospheric carbon dioxide (CO_2_) concentration has risen from the pre-industrial level of approximately 280 ppm to approximately 414 ppm in 2020 [115] and is expected to reach a maximum of 600 ppm in 2050 [99]. The effects of elevated CO_2_ concentrations on soil microorganisms are likely to be mediated by increased photosynthetic carbon production and input of withered branches and fallen leaves to the soil, as well as root secretion and nutrient effectiveness [104,116], increased microbial as well as plant activity, and increased likelihood of heavy metals being adsorbed into the food chain. Ocean acidification is a marine environmental problem caused by greenhouse gas emissions [117], and seawater acidification caused by the increase in carbon dioxide content in the marine environment may cause the migration of heavy metals from the sediment surface to the seawater column [118]. pH is the most influential factor in the movement and storage of heavy metals, as it directly affects the adsorption/desorption reactions and ion exchange on the surface, and indirectly impacts the dissolution/precipitation of organic matter and hydroxides, the dissolution of carbonates, and the dissolution of sulfides [119]. Ocean acidification exacerbates the loss of marine biodiversity, especially in the tropics [120], as well as changes in the polar migration of species and the interruption of the biology and physiology of marine species [121]. With an increase in temperature and a decrease in pH value, the solubility of heavy metals in the aqueous phase increases and the content of heavy metals in sediments decreases [51], affecting biological and ecotoxicological reactions and increasing the movement of these metals [122].

### 3.3. Organic Matter

Heavy metals are often found in higher concentrations in marine or lake sediment than in the water column, resulting in a hazard to the benthic biota [123] and leading to a restructuring of the sediment microbial communities [124]. Temperature and precipitation both have an influence on the deposition of heavy metals, nutrients, and organic matter. Organic matter is full of various functional groups and binding sites; thus, heavy metals can be found in aquatic environments in the form of ions or attached to negatively charged organic or inorganic components [125]. It is evident that organic matter has a major impact on the adsorption and sedimentation of heavy metals in the bottom sediments of these lakes, as evidenced by the strong correlation between heavy metals and lake nutrients. Global warming raises temperature, and this rise in temperature enhances microbial metabolism, which promotes microbial abundance and thus promotes the decomposition of organic matter [126], thus affecting the deposition of heavy metals. A rise in average temperature may also promote secondary emissions of heavy metals [94], and the heavy metals deposited at the bottom of the ocean or lake are released and diffuse into the overlying water.

### 3.4. Soil Moisture

A rise in global climate temperature will increase the intensity and duration of drought, but when the temperature continues to rise to a certain level, atmospheric water vapor will increase, producing more intense precipitation events [127], which can lead to a drastic change in soil moisture. The soil microbiome’s response to other disturbances, such as heat waves, freeze–thaw cycles, and pollution, is determined by the changes in soil and microbial properties that occur after drying and rewetting, a problem that has become increasingly prevalent in many soil ecosystems [128]. Drying and rewetting increase the inhibitory effect of heavy metals on enzyme activity in chronically contaminated soils, because the decomposition of soil aggregates can release available heavy metals [129]. As air drying causes soil to dry up, the resistance of soil enzymes decreases due to their increased exposure to heavy metals. Heavy metal toxicity sensitivity of soil enzymes is significantly impacted by the climate and soil properties when drying and rewetting occur [130].

## 4. Effects of Heavy Metals on Organisms under Global Warming

**Figure 2:**
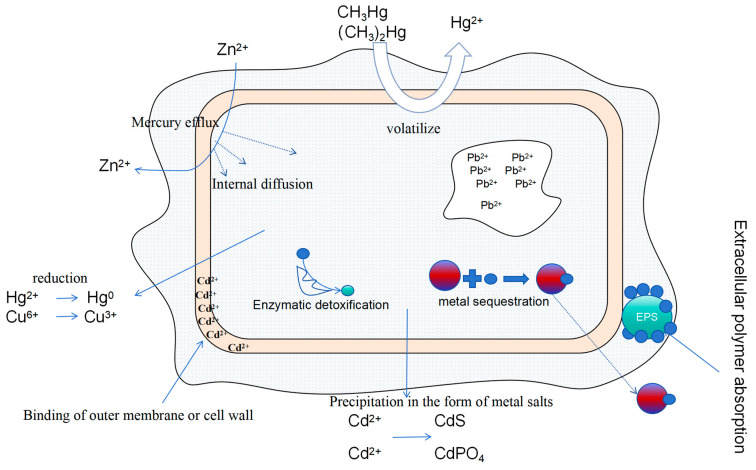
Translational pathways of heavy metal transfer in cells.

The rise in temperature, changes in precipitation, and rise in carbon dioxide concentration caused by global warming amplify the impacts of heavy metal pollutants on ecosystems [131]. Human activities have caused heavy metals to be released into the environment, and they are more easily deposited under global warming. Heavy metals are imported into various ecosystems through atmospheric particulate deposition and other pathways, and then passed through the food chain to inhibit the growth of organisms at the back end of the biological chain, thus breaking some of the biological balances of the ecosystems [132,133]. Figure 2 is a schematic diagram of the translocation transformation pathway of heavy metals in the cellular fraction [134].

### 4.1. Aquatic Life

Heavy metals are a major environmental stressor for marine organisms [135], and toxic heavy metals reduce the upper limit of thermal tolerance of polluted aquatic environments. An increase in temperature will continue to bring new thermal challenges to aquatic animals. With the temperature continuing to go up, aquatic animals will be presented with new thermal issues [136]. Research indicates that when temperature and heavy metals interact, the result is a disruption of mitochondrial performance, oxidation, an accumulation of lipid peroxidation products, and harm to lysosomes and DNA [137]. Phytoplankton can absorb toxic heavy metals, and these metals can then be transferred up the food chain, thus posing a risk to human health.

Morphological defense is considered an effective anti-herbivory strategy of phytoplankton [138]. Some studies have shown that heavy metal ions reduce the maintenance time of morphological defense by interfering with the formation of semiotic substances. The increase in temperature reduced the size of induced defense colonies while enhancing the toxicity of heavy metal ions [139], promoting the inhibitory effect of the heavy metal ions on the formation of induced colonies of Streptococcus obliquus, and the two factors worked together to produce a more severe effect on the induced defense [140].

Some studies have shown that global warming and heavy metal pollution pose certain threats to primary producers, such as algae. Warming significantly increases the production of reactive oxygen species in algae and affects the metabolism of various amino acids [141]. Some studies have shown that nickel enrichment at ambient temperature can lead to an increase in the calcification rate of corals [142]. However, when the temperature rises, the synergistic effect of nickel enrichment and temperature on corals is negative. Under the influence of long-term and moderate nickel input and warming, the growth rate of coral communities has decreased by 37% [142].

Diatoms that have long-term adaptations to ocean acidification exhibit significantly lower growth and photosynthesis, increasing the sensitivity of marine diatoms to toxic heavy metal exposure [143]. Elevated temperatures will promote the growth of algae [144], thereby increasing the pH of the water [145,146]. The combined effects of pH and temperature, and the toxicity of copper on microalgae have been studied using acclimatized cells, and ecotoxicity studies have shown that increasing temperature (20–30 °C) and pH make copper more toxic to these microalgae. Elevated water temperature enhances cell membrane permeability and metabolism [147], and this change can increase the uptake of nutrients and heavy metals by algae. The speciation and toxicity of heavy metals are affected by the pH. Different forms of heavy metals have different effects on the toxicity of algae. A high pH value controls the bioavailability and toxicity of copper [148], and a variety of heavy metals are less toxic at low pH values, which may be due to the competition between hydrogen ions and heavy metal ions for cell surface binding sites or ligands [149].

The thermal induction of Hsp70 in bivalves is significantly reduced due to heavy metals, resulting in a decrease in their aerobic capacity. Mitochondrial dysfunction in oysters exposed to temperature and heavy metal stress causes cellular energy deficiency, thus limiting the ATP available for the synthesis or functioning of Hsp [137]. Elevated cadmium concentrations in the digestive glands and gills of mussels exposed to heavy metals were exacerbated by co-exposure to higher temperatures, and interactions between temperature and pH on Cd-mediated induction of heavy metal thionein, antioxidant system reactivity, and the onset of oxidative damage to lipids were noted, with a tissue-specific effect [150]. Cadmium and temperature affect phagocytosis efficiency and the blood cell population composition. They may affect the micronucleus frequency through different mitosis rates. During the warm period, bivalves had a low tolerance to estuarine pollution and reproduced in the intertidal environment. Periods with tides and seasonal temperature fluctuations, as well as global climate change, have posed problems for their survival [151,152].

Under global warming, the heart rate of zebrafish embryos was observed to have risen only when heat stress was present. Furthermore, when exposed to both heavy metals and heat stress, the embryos showed an abnormal morphology with a curved body shape [153]. In addition, heat stress also triggers the copper-induced intracellular production of reactive oxygen species (ROS), causing toxic effects in fish [154,155]. The effect of heavy metal contamination on the transcription of genes linked to the antioxidant system, metabolism, inflammation, cell apoptosis, and DNA methylation was enhanced by heat stress [153,156,157,158,159].

Exposure to copper, a heavy metal, at levels that result in systemic concentrations has a drastic effect on the heat tolerance of salmon flies, possibly because it disrupts oxygen uptake through respiratory surfaces, transport within the body, or utilization in mitochondria [160]. The ability of sea urchins to cope with heavy metals has decreased over the past two decades due to changes in environmental variables [161]. The mercury concentrations in the teeth of modern beluga whales were, on average, three to five times higher than those in historical beluga whales. Similar ecological feeding changes in different regions and species suggest that, with the gradual warming of the Arctic Ocean and the disappearance of sea ice, the adverse response to climate change of the upper layers of the food web continues to increase [162,163,164].

### 4.2. Soil Organisms

**Figure 3:**
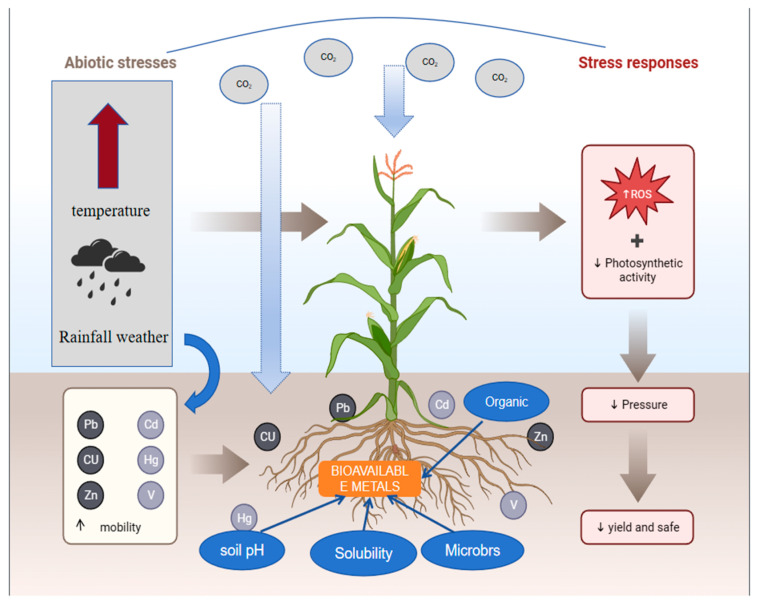
Climate-driven impacts of heavy metals on crops. Note: ↑ indicates an increase, ↓ indicates a decrease.

The main environmental source of heavy metals for plant uptake is soil, which is impacted by atmospheric deposition, livestock manure, wastewater irrigation, heavy metal pesticides or herbicides, phosphate-based fertilizers, and sewage sludge-based amendments in the agricultural setting [165]. High concentrations of heavy metals retard microbial community growth by disrupting cell division, transcription, and translation, or by disrupting membranes and denaturing DNA and proteins [166]. Soils with high levels of heavy metals can cause soil fertility to suffer, leading to physiological disorders, disrupting plant metabolism, and resulting in stunted growth and decreased yields [167]. Figure 3 is a schematic representation of the effects of heavy metals on crops under climate-driven conditions.

The interaction between global climate change and heavy metals destroys the function and activity of enzymes by replacing essential heavy metals and nutrients [168], leading to oxidative stress in plants and thus impacting agricultural crops, affecting crop growth and development, and having a direct impact on yield and food safety [169].

The ecotoxicity of soils contaminated with heavy metals is usually related to the bioavailability of the heavy metals present in the system [170], as well as the climatic conditions under which soil organisms are exposed [171]. Climatic conditions may alter heavy metal biogeochemistry/species formation through changes in soil properties [172]; however, they also induce physiological stresses in the soil biota, which can alter the sensitivity of organisms to heavy metals [173]. Therefore, the heavy metal toxicity of soil ecology may vary according to the current climatic conditions. Climate change may increase the toxic effect of heavy metal-contaminated field soils on invertebrates. One study showed that while exposure to heavy metal-contaminated soil did not have a harmful effect on the performance of Candida, climate change can make heavy metals toxic to Candida, especially as the temperature increases [174]. Copper exposure will affect the membrane phospholipids of springtails, which will eventually affect the membrane fusion process of cells and reduce the heat tolerance of springtails [175].

Elevated atmospheric carbon dioxide concentration increased the number and weight of a certain soybean variety, while heavy metal pollution reduced this growth and killed some seeds [176]. The joint effect of drought and heavy metals reduces the sensitivity of trees (the ability to respond to stress), and heavy metal pollution has a great impact on reducing the sensitivity of trees to climatic factors [177]. Table 3 lists the heavy metal accumulation of organisms in different environments.

## 5. Conclusions and Prospects

The current study preliminarily proves that global warming has some impacts on heavy metals in the environment. Heavy metal migration causes shifts in heavy metal contents in the environment, and these changes in the speciation of heavy metals result in modifications in the toxicity and bioavailability of heavy metals in the ecosystem. The movement of heavy metals causes changes in the amount of heavy metals present in the environment, and these shifts in the speciation of heavy metals can cause differences in the toxicity and bioavailability of heavy metals in the ecosystem. In most studies, this effect is negative. The most direct impact of temperature rise is the melting of alpine or polar glaciers, which transfers the heavy metals contained in them to the environment. When the elements are released, the content of heavy metals increases, and the extent to which organisms are affected increases. Weather and climate change, including extreme rainfall, accelerate the migration process of heavy metals. When the carbon dioxide concentration increases, the pH of the aquatic environment increases, and the form of heavy metals changes as a result. Many studies show that these changes increase the toxicity of heavy metals and enhance the adverse effects of heavy metals on ecosystems.

The impact of heavy metals on ecosystems is long-term. To better address the adverse effects of heavy metals on ecosystems and further make up for the knowledge gap between global climate and environmental change and environmental heavy metals, it is necessary to investigate the impact of heavy metals in various types of ecosystems. Longer-term field experiments are conducted to explore the biogeochemistry between long-term climate change and heavy metals and to look for more ecologically healthy ways of adsorption and degradation of heavy metals; for example, using biological materials to degrade and adsorb heavy metals and using microorganisms to reduce the impact of heavy metals on ecosystems. In the future, we also hope that methods can be found to block the influence of climate on the contents and forms of heavy metals.

## Figures and Tables

**Table 1:** Changes in heavy metal contents in oceans and lakes.

Location	HM (ppm)							References
	Cr	Co	Cu	Pb	Cd	Ni	Zn	
Kongsfjorden, Arctic (1925–2018)	29.27–234.18	14.091–77.24	16.05–401.75	6.83–98.98	0.14–1.05	31.1–178.82	46.48–190.62	[35,36,37]
Tibetan Plateau (1836–2014)	85.73–1244		60.07–118.6	20.36–37.85	0.13–0.44	52.65–335.6	80.58–168.7	[38]
Renuka Lake (1839–2003)	5–62	7–51	11–40	21–27		11–33	43–127	[39]
Poyang lake (1950–2005)				48.30–54.53	0.23–0.59			[40]
Romanian Black Sea (1996–2012)	1.29–15.01		19.36–96	0.15–27.70	0.45–14.5	20–106.34		[41]

**Table 2:** Environmental heavy metal contents in different regions.

Location	Sample	HM							Reference
		Cu	Pb	Cd	Zn	Cr	Ni	Hg	
South Durban beaches	Sediments (ppm)	50.53	14.00	0.70	64.56	302	33.32	0.98	[78]
South Durban beaches	Sediments (ppm)	33.63	16.06	0.41	44.57	298	101.98		[79]
Suez Gulf, Egypt	Coastal sediments (ppm)	13.73	49.25	5.8	48.59	12.89	53.59		[80]
South Australian coastline	Coastal sediments (ppm)	50.22	662	22.12	1609	26.62	662		[81]
Spain	Coastal sediments (ppm)	31.8	22.2	0.30	70.2	18.5	13.8		[82]
Bahia Solano Beaches, Colombia	Coastal sediments (ppm)	53.93	409.67		125.80	269.17			[83]
Acapulco beach, Mexico	Coastal sediments (ppm)	0.76–26.97	0.13–20.46	0.10–12.51	3.54–96.23	0.64–105.50	0.33–16.35		[84]
Marmara Sea, Turkey	Coastal sediments (ppm)	20	25.4	0.50	43	47.8			[85]
Nepal	Surface soil (ppm)	132–1010	59.8–294	27.2–93	606–4260	135–393	606–4260		[86]
European mountain beech forests	Soils (ppm)	11.3–39.8	1.38–91.8	0.99–6.03	32.5–252	4.22–83.4	4.79–56.3	32.5–252	[87]
Kuril-Kamchatka Trench	Surface sediments (ppm)	86.2	16.9		86.3		38.2	76 (ppb)	[88]
Bering Sea	Surface sediments (ppm)	36.4	8.7		100		34.6	55 (ppb)	
Sea of Okhotsk	Surface sediments (ppm)	47.6	18.6		97.80		41.6	88 (ppb)	
Shenzhen	Soils (ppm)	57.46 ± 0.73	72.96 ± 0.87	2.24 ± 0.33	234.82 ± 15.61	110.43 ± 0.94	37.98 ± 0.16	0.46 ± 0.06	[89]
Nigeria	Water samples (ppm)	0.03–0.30	0.02–0.08	0.02–0.08 *	0.55–1.33 *	0.03–0.30 *	0.01–0.04 *		[90]
Mt. Gongga (3600–3700 m)	Soils (ppm)	133.5	17.9	0.2	47.8	133.5	17.9	56.6 (ppb)	[91]
Natal	RDS, DS, RS (ppm)	26.9–108.4	49.7–119.4	3.2–4.8	31.8–149.8	19.5–33.7			[92]
Beijing	Soils (ppm)			0.12	26.8	29.8	26.8		[93]
China	Sediment of lake (ppm)	33.93 ± 5.21–64.91 ± 3.16	20.49 ± 1.68–30.83 ± 0.55	0.25 ± 0.05–0.39 ± 0.08	120.17 ± 10.25–128.86 ± 6.69	87.83 ± 4.81–100.33 ± 10.04	44.16 ± 2.97–62.87 ± 3.83	23.25 ± 3.16–76.00 ± 4.31 (ppb)	[94]
Yangxin County	Soils (ppm)	144.9 *	69.4	2.9 *	188.3	55.5	137.0 *		[95]
Morocco	Soils (ppm)	36.65 ± 2.63	53.25 ± 3.23	14.2 ± 0.81	85.21 ± 2.42	27.33 ± 1.58			[96,97]
India	Groundwater (ppb)	56.30	29.75 *	12.94	800	102.94 *	40.60		[71]
India	Seawater (ppb)	2022.7 *	30.42 *	5.70	252.9	1118.5 *	450 *		
Odisha coastal plains	groundwater (ppm)	4.09 ± 1.73		0.05 ± 0.14	1.46 ± 2.44	0.28 ± 0.83	1.46 ± 2.44		[98]
Pakistan	groundwater (ppm)	0.09–2.63	0.0–1.05	0.0–0.94	0.09–2.63				[99]
Guangzhou	Soil (ppm)	56.81	50.67		131.33	77.20	38.31	0.19	[100]

*: Exceeded the allowable limit in the standards.

**Table 3:** Accumulation of heavy metals in organisms in different environments.

Area	Organism	HM (ppm)								References
		Cu	Hg	Cd	Cr	Pb	Ni	Co	Zn	
Bay of Bengal	Zooplankton	84.9 ± 6.7		46.2 ± 5.6		19.2 ± 2.6	62.8 ± 6.5	46.2 ± 4.6		[178]
Antarctica	Algae	0.30–36.70		1.06–3.34	1.72–29.85	0.26–3.15	1.32–19.13			[179]
southeast coast of China	rice	1.170–26.616 *	0.002–0.063 *	0.000–0.684 *	0.001–0.897	0.008–0.302 *	0.013–0.884 *			[180]
Kali River	crops	3.8–42.7		0.06− 0.69 *	0.23–90.7	7.05–65.05	19–29.4		12.9–86.2	[181]
Grand Forest Park	leaf	0.10–0.54	0.12–0.14	2.35–3.60						[182]
The Beibu Gulf	Charybdis miles	29.94 ± 3.02		569.9 ± 541.0 (ppb)	230.7 ± 181.3 (ppb)	52.1 ± 4.9 (ppb)	118.6 ± 72.6 (ppb)		123.86 ± 39.85	[183]
Laizhou Bay	Aquatic organisms	19.60		0.40	0.39	0.25	0.51		60.11	[184]

*: Heavy metal content of some samples exceeded the allowable limits of some standards.

## Data Availability

All data analyzed during this study are included in this article.
